# Enhanced expression of mRNA for nuclear factor κB1 (p50) in CD34+ cells of the bone marrow in rheumatoid arthritis

**DOI:** 10.1186/ar1915

**Published:** 2006-03-06

**Authors:** Shunsei Hirohata, Yasushi Miura, Tetsuya Tomita, Hideki Yoshikawa, Takahiro Ochi, Nicholas Chiorazzi

**Affiliations:** 1 Department of Internal Medicine, Teikyo University School of Medicine, Tokyo 173-8605, Japan; 2 Department of Rheumatology, Kobe University FHS School of Medicine, Kobe 654-0142, Japan; 3 Department of Orthopedic Surgery, Osaka University Medical School, Osaka 565-0871, Japan; 4 Sagamihara National Hospital, Kanagawa 228-8522, Japan; 5 Experimental Immunology and Rheumatology, North Shore-LIJ Research Institute, Manhasset, NY 11030, USA

## Abstract

Bone marrow CD34+ cells from rheumatoid arthritis (RA) patients have abnormal capacities to respond to tumor necrosis factor (TNF)-α and to differentiate into fibroblast-like cells producing matrix metalloproteinase (MMP)-1. We explored the expression of mRNA for nuclear factor (NF)κB in RA bone marrow CD34+ cells to delineate the mechanism for their abnormal responses to TNF-α. CD34+ cells were purified from bone marrow samples obtained from 49 RA patients and 31 osteoarthritis (OA) patients during joint operations via aspiration from the iliac crest. The mRNAs for NFκB1 (p50), NFκB2 (p52) and RelA (p65) were examined by quantitative RT-PCR. The expression of NFκB1 mRNA in bone marrow CD34+ cells was significantly higher in RA than in OA, whereas there was no significant difference in the expression of mRNA for NFκB2 and RelA. The expression of NFκB1 mRNA was not correlated with serum C-reactive protein or with the treatment with methotrexate or oral steroid. Silencing of NFκB1 by small interfering RNA abrogated the capacity of RA bone marrow CD34+ cells to differentiate into fibroblast-like cells and to produce MMP-1 and vascular endothelial growth factor upon stimulation with stem cell factor, granulocyte-macrophage colony stimulating factor and TNF-α without influencing their viability and capacity to produce β2-microglobulin. These results indicate that the enhanced expression of NFκB1 mRNA in bone marrow CD34+ cells plays a pivotal role in their abnormal responses to TNF-α and, thus, in the pathogenesis of RA.

## Introduction

Rheumatoid arthritis (RA) is a chronic inflammatory disease characterized by hyperplasia of synovial lining cells, consisting of macrophage-like type A synoviocytes and fibroblast-like type B synoviocytes [1]. It has been appreciated that type A synoviocytes, which are also called intimal macrophages, are derived from monocyte precursors in the bone marrow [1]. On the other hand, type B synoviocytes, which are also called fibroblast-like synoviocytes, have the morphological appearance of fibroblasts as well as the capacity to produce and secrete a variety of factors, including proteoglycans, cytokines, arachidonic acid metabolites, and matrix metalloproteinases (MMPs), that lead to the destruction of joints [1]. Apart from type A synoviocytes, the origin of type B synoviocytes has been unclear [1]. Of note, we have recently demonstrated that bone marrow CD34+ cells from RA patients have abnormal capacities to respond to tumor necrosis factor (TNF)-α and to differentiate into fibroblast-like cells producing MMP-1, suggesting that bone marrow CD34+ progenitor cells might generate type B synoviocytes and thus could play an important role in the pathogenesis of RA [2].

TNF-α is one of the first triggers to be found effective for the activation of nuclear factor (NF)κB in RA synovium [3]. This mechanism of activation was followed by up-regulation of several inflammatory genes usually found in active RA. Accordingly, a number of studies have shown that TNF-α blockade has beneficial effects in the treatment of RA [4]. Moreover, inhibition of NFκB by the antioxidant N-acetylcysteine significantly reduced TNF-α- and NFκB-dependent gene expression and synovial proliferation [3]. We thus hypothesized that abnormal responses of RA bone marrow CD34+ cells to TNF-α might result from abnormal expression of NFκB genes. The current studies were undertaken, therefore, to explore the expression of mRNA for various components of NFκB in bone marrow CD34+ cells in RA.

## Materials and methods

### Patients and samples

Bone marrow samples were obtained from 49 patients with RA (8 males and 41 females: mean age, 58.6 years; age range, 35 to 78 years) who satisfied the American College of Rheumatology 1987 revised criteria for RA [5] and gave informed consent in accordance with the World Medical Association Declaration of Helsinki Ethical Principles for Medical Research Involving Human Subjects. The samples were taken during joint operations via aspiration from the iliac crest under anesthesia. As a control, bone marrow samples were similarly obtained from 31 patients with osteoarthritis (OA; 3 males and 28 females; mean age, 71.2 years; age range, 49 to 81 years) who gave informed consent. Most patients with RA and OA were taking non-steroidal anti-inflammatory drugs. Of the 45 patients with RA, 23 were treated with low dose methotrexate (MTX) and 33 were taking oral steroids when bone marrow samples were obtained. No OA patients were taking MTX or oral steroid.

### Preparation of bone marrow CD34+ cells

Mononuclear cells were isolated by centrifugation of heparinized bone marrow aspirates over sodium diatrizoate-Ficoll gradients. CD34+ cells were purified from the mononuclear cells by positive selection with magnetic beads (CD34 progenitor cell selection system; Dynal, Oslo, Norway). The cells thus prepared were >95% CD34+ cells and <0.5% CD19+ B cells, as previously described [2]. In addition, CD34+ cells derived from bone marrow aspirates from the iliac crests of healthy individuals (purity >95%) were purchased from BioWhittaker (Walkersville, MD, USA).

### RNA isolation and real-time quantitative PCR

Total RNA was isolated from purified bone marrow CD34+ cells using the Trizol reagent (Life Technologies, Grand Island, NY, USA) according to the manufacturer's instructions. cDNA samples were prepared from 1 μg of total RNA using the SuperScript reverse transcriptase preamplification system (Life Technologies) with oligo (dT) primer and subjected to PCR. Real-time quantitative PCR was performed using the LightCycler rapid thermal cycler system (Roche Diagnostics, Lewes, UK) with primer sets for NFκB1, NFκB2, RelA or β-actin and Light Cycler-Fast Start DNA master SYBR Green I (Roche Diagnostics). The primers were designed using Oligo Primer Analysis Software version 5.0 (Takara Bio Inc., Ohtsu, Japan). The detail of primer sequences is shown in Table 1. Quantitative analysis was performed using LightCycler Software v.3.5. PCR reaction conditions composed of denaturing at 95°C for 10 minutes for 1 cycle, followed by 40 cycles of denaturing (10 seconds at 95°C), annealing (10 seconds at 60°C (NFκB2, RelA) or 62°C (NFκB1, β-actin)), and extension (5 seconds (NFκB1), 6 seconds (NFκB2, RelA), or 10 seconds (β-actin) at 72°C).

### Immunofluorescence staining and analysis

Purified bone marrow CD34+ cells (obtained from three RA patients and three OA patients) were treated with IntraPrep™ Permeabilization Reagent (Immunotech, Marseille, France), followed by staining with phycoerythrin (PE)-conjugated anti-NFκB p50 (E-10; a mouse IgG1 monoclonal antibody against amino acids 120 to 239 mapping at the amino terminus of human NFκB p50; Santa Cruz Biotech, Santa Cruz, CA, USA) or PE-conjugated normal mouse IgG1 (Santa Cruz). The cells were analyzed using an EPICS XL flow cytometer (Coulter, Hialeah, FL, USA) equipped with an argon-ion laser at 488 nm. A combination of low-angle and 90° light scatter measurements (forward scatter versus side scatter) was used to generate a bit map gating to identify bone marrow cells using Cyto-Trol™ Control Cells (Coulter) and Immuno-Trol™ Cells (Coulter) as standards. Specific mean fluorescence intensity (MFI) for NFκB1 (p50) was calculated by subtracting the non-specific MFI of staining with the isotype-matched control mouse IgG1.

### Culture medium and cytokines

RPMI 1640 medium (Life Technologies) supplemented with L-glutamine (0.3 mg/ml) and 10% fetal bovine serum (Life Technologies) was used for all cultures. Recombinant human stem cell factor (SCF), granulocyte-macrophage colony stimulating factor (GM-CSF), and TNF-α were purchased from Pepro Tech EC (London, UK).

### Silencing of NFκB1 in bone marrow CD34+ cells by small interfering RNA

SMARTpool^® ^small interfering RNA (siRNA) for NFκB1 (p50) gene and nonsense scrambled control siRNA were purchased from Dharmacon (Lafayette, CO, USA). Chemical transfection of siRNAs into bone marrow CD34+ cells was performed using siPORT™ Amine Transfection Agent (Ambion, Austin, TX, USA) according to the manufacturer's directions. Briefly, purified bone marrow CD34+ cells were cultured in a 24-well microtiter plate (N0. 3524; Costar, Cambridge, MA, USA) at 2 × 10^5 ^cells per well in 0.2 ml culture medium in the presence of SCF (10 ng/ml) and GM-CSF (1 ng/ml). After 24 hours of incubation, chemical transfection of siRNAs was performed, and incubated for 4 hours.

### Cell cultures and measurement of MMP-1 and vascular endothelial growth factor

After transfection of siRNAs, the cells were cultured with SCF (10 ng/ml) and GM-CSF (1 ng/ml) in 1.0 ml culture medium for 48 hours and then harvested for RNA extraction. Alternatively, the cells were cultured in a 24-well microtiter plate at 2 × 10^5 ^cells per well in 1.0 ml culture medium for 4 weeks in the presence of SCF (10 ng/ml), GM-CSF (1 ng/ml) and TNF-α (10 ng/ml) without medium change, as previously described [2]. The differentiation of fibroblast-like cells was observed under the phase-contrast light microscopy. The concentrations of MMP-1 and vascular endothelial growth factor (VEGF) in the culture supernatants were measured using the Biotrak human MMP-1 ELISA system (Amarsham Pharmacia Biotech, Buckinghamshire, UK) and human VEGF immunoassay kit (BioSource International, Camarillo, CA, USA), respectively. The concentrations of β_2_-microglobulin (β_2_MG) were determined by an ELISA as previously described [6].

### Statistics

Comparison between RA and OA patients and between RA patients with MTX or steroid and those without MTX or steroid was carried out using Welch's *t* test. Significance of the effects of siRNA transfection on the generation of fibroblast-like cells and on the production of MMP-1 and VEGF was evaluated by Wilcoxon's signed rank test. Correlation between serum C-reactive protein and NFκB1 mRNA in bone marrow CD34+ cells and that between NFκB1 mRNA and protein were evaluated using a liner regression test. Correlation between NFκB1 mRNA in bone marrow CD34+ cells and the generation of fibroblast-like cells was analyzed using a Spearman's rank correlation test.

## Results

### Expression of mRNAs for various components of NFκB in bone narrow CD34+ cells

**Figure 1:**
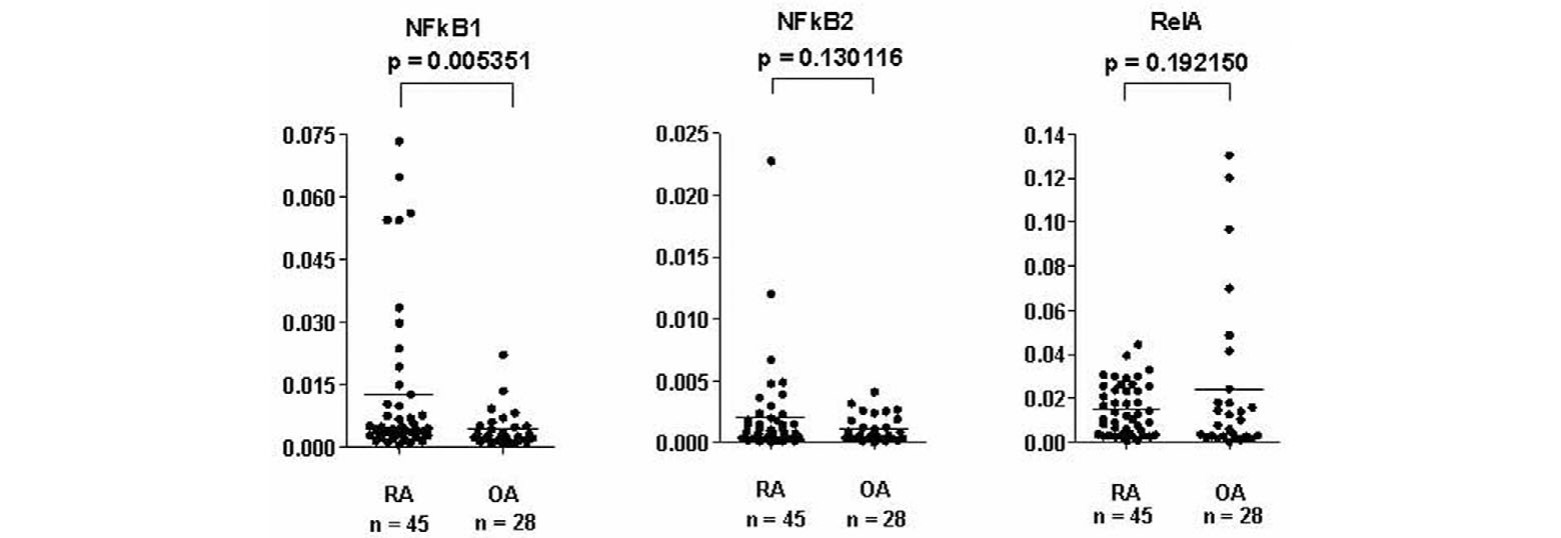
The expression of mRNAs for nuclear factor (NF)κB1 (p50), NFκB2 (p52) and RelA (p65) in bone marrow CD34+ cells. Total RNA was isolated from purified bone marrow CD34+ cells. The expression of mRNAs for NFκB1, NFκB2, RelA and β-actin was evaluated by real-time quantitative PCR. The data are expressed as the ratio of the mRNA copy numbers to those of β-actin. Horizontal lines indicate the mean values. Statistical significance was evaluated by Welch's *t* test. OA, osteoarthritis; RA, rheumatoid arthritis.

The expression of mRNA for NFκB1 (p50), NFκB2 (p52), and RelA (p65) in bone marrow CD34+ cells is shown as the ratio of the copy numbers to those of β-actin mRNA in Figure 1. The expression of NFκB1 mRNA was significantly higher in RA bone marrow CD34+ cells than in OA bone marrow CD34+ cells (*p* = 0.005351), whereas there were no significant differences in the expression of NFκB2 mRNA (*p* = 0.130116). Although the expression of RelA mRNA appeared to be lower in RA bone marrow CD34+ cells than in OA bone marrow CD34+ cells, it did not reach statistical significance (*p* = 0.192150). These results indicate that the expression of mRNA for components of NFκB1 is exclusively enhanced in bone marrow CD34+ cells from patients with RA.

**Figure 2:**
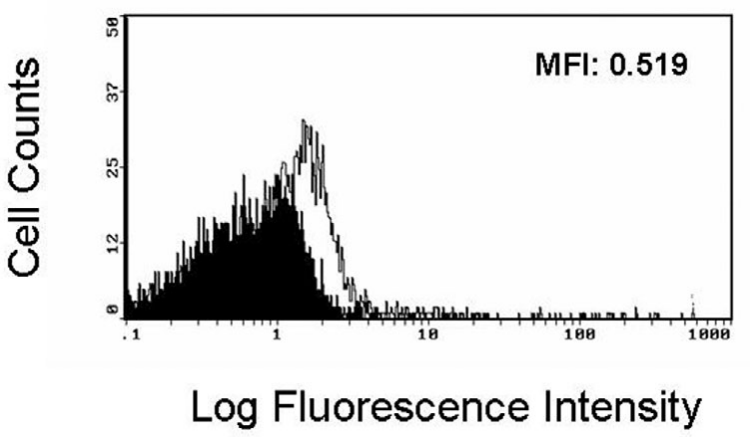
Expression of nuclear factor (NF)κB1 (p50) protein in bone marrow CD34+ cells. Purified bone marrow CD34+ cells from a rheumatoid arthritis patient were permeabilized and then stained with phychoerythrin-conjugated anti-NFκB p50 monoclonal antibody or phychoerythrin-conjugated normal mouse IgG1, followed by analysis with flow cytometry. The level of NFκB1 protein was expressed by mean fluorescence intensity as described in Materials and methods.

**Figure 3:**
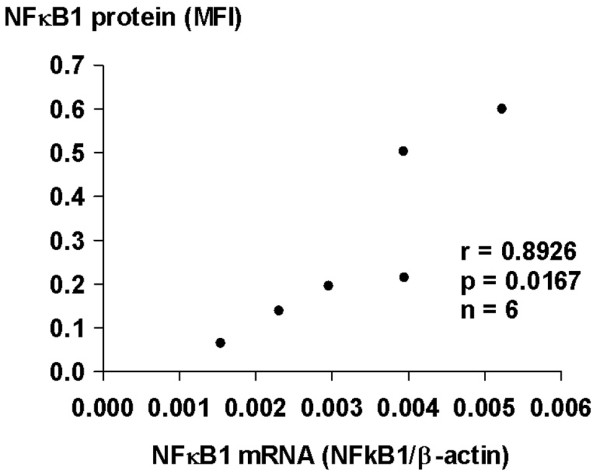
Comparison of the expression of nuclear factor (NF)κB1 (p50) protein with that of NFκB1 mRNA in bone marrow CD34+ cells. Purified bone marrow CD34+ cells were permeabilized and then stained with phychoerythrin-conjugated anti-NFκB p50 monoclonal antibody or phychoerythrin-conjugated normal mouse IgG1, followed by analysis with flow cytometry. The NFκB1 protein levels as expressed by mean fluorescence intensity were compared with NFκB1 mRNA levels (expressed as the ratio of the mRNA copy numbers to those of β-actin) in bone marrow CD34+ cells from six patients (three rheumatoid arthritis patients and three osteoarthritis patients). Statistical significance was evaluated by linear regression test.

Next, experiments were carried out to examine whether the elevation of NFκB1 mRNA expression parallels the elevation of NFκB1 protein expression in bone marrow CD34+ cells. The protein expression of NFκB1 was evaluated by staining of permeabilized bone marrow CD34+ cells from three RA patients and three OA patients with anti-NFκB p50 monoclonal antibody, followed by analysis with flow cytometry. As can be seen in Figure 2, bone marrow CD34+ cells express NFκB1 (p50) protein, the quantity of which can be expressed as MFI. Moreover, there is significant correlation between MFI for NFκB1 and NFκB1 mRNA in the six bone marrow CD34+ cells (Figure 3). The results indicate that the elevation of NFκB1 mRNA leads to the increase in NFκB1 protein expression.

### Relevance of expression of NFκB1 mRNA in bone marrow CD34+ cells from RA patients to treatment and clinical parameters

**Figure 4:**
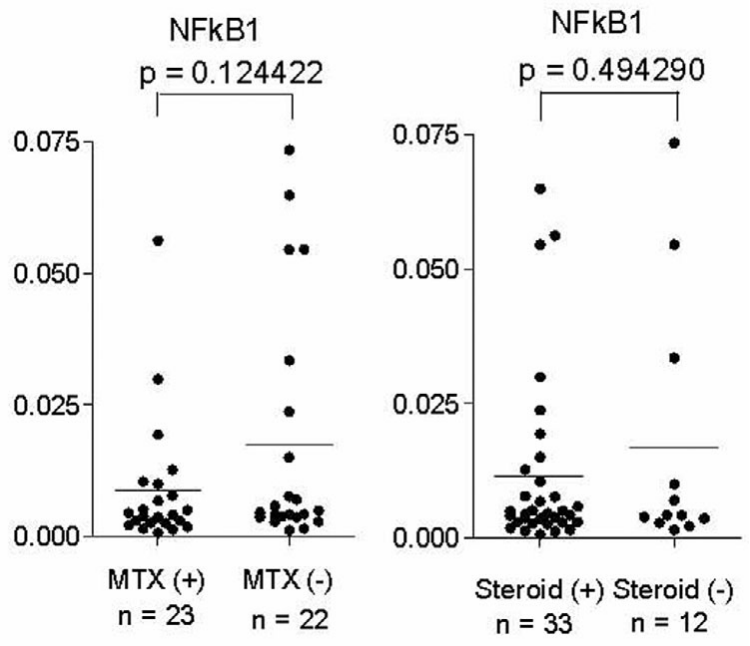
The relevance of treatment with the expression of mRNAs for nuclear factor (NF)κB1 mRNA in bone marrow CD34+ cells. Total RNA was isolated from purified bone marrow CD34+ cells from 45 rheumatoid arthritis patients. The expression of mRNAs for NFκB1 and β-actin was evaluated by real-time quantitative PCR. The data are expressed as the ratio of the mRNA copy numbers to those of β-actin. Effect of treatment with methotrexate (MTX) or oral steroids (Steroid) was evaluated by Welch's *t* test. Horizontal lines indicate the mean values.

**Figure 5:**
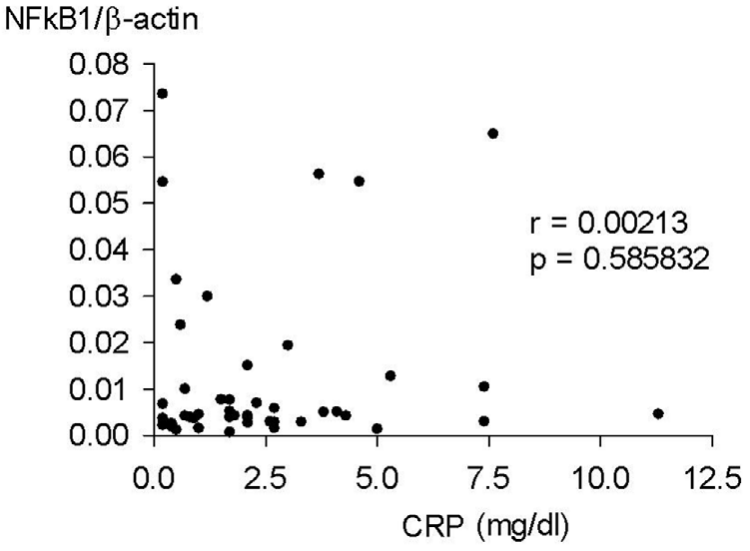
The correlation of the expression of mRNAs for nuclear factor (NF)κB1 mRNA in bone marrow CD34+ cells with serum C-reactive protein (CRP). Total RNA was isolated from purified bone marrow CD34+ cells from 45 rheumatoid arthritis patients. The expression of mRNAs for NFκB1 and β-actin was evaluated by real-time quantitative PCR. The data are expressed as the ratio of the mRNA copy numbers to those of β-actin. Statistical significance was evaluated by linear regression test.

Of note, 22 and 33 of the 45 RA patients were treated with MTX and oral steroids, respectively, whereas no OA patients were taking either MTX or oral steroids. It is therefore possible that MTX and oral steroids might have affected the expression of NFκB1 mRNA in bone marrow CD34+ cells. As shown in Figure 4, however, there were no significant differences in the expression of NFκB1 mRNA in bone marrow CD34+ cells between RA patients taking MTX or oral steroids and those who were not, although the expression of NFκB1 mRNA appeared to be lower in RA patients taking MTX or oral steroids. It is unlikely, therefore, that the medication the RA patients were taking would have resulted in the upregulation of NFκB1 mRNA expression in bone marrow CD34+ cells. It should be also noted that the expression of NFκB1 mRNA in bone marrow CD34+ cells was not significantly correlated with serum C-reactive protein (CRP) levels in RA patients (Figure 5). The data thus indicate that the upregulation of NFκB1 mRNA in bone marrow CD34+ cells is independent of the activity of the systemic inflammation, as reflected by serum CRP.

### Relevance of the expression of NFκB1 mRNA to the generation of fibroblast-like cells

**Figure 6:**
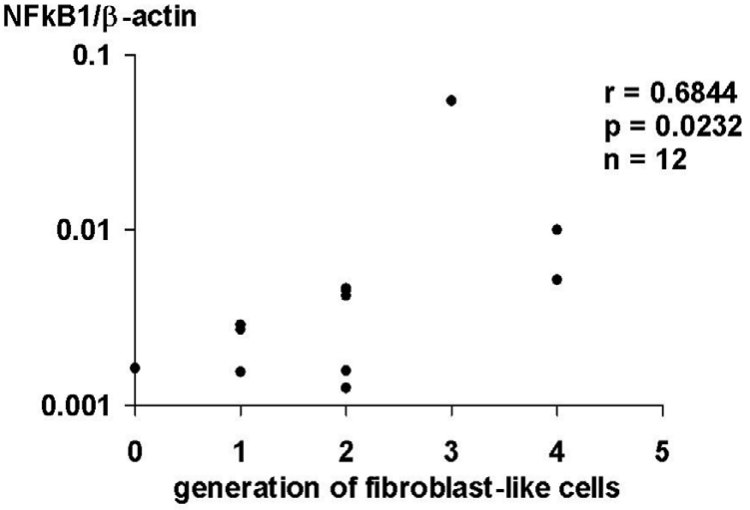
Comparison of the expression of nuclear factor (NF)κB1 (p50) mRNA in bone marrow CD34+ cells with their capacity to differentiate into fibroblast-like cells. The expression of NFκB1 mRNA in bone marrow CD34+ cells from 12 rheumatoid arthritis patients was evaluated by real-time quantitative PCR prior to the culture. The bone marrow CD34+ cells were incubated in culture medium with stem cell factor (10 ng/ml), granulocyte-macrophage colony stimulating factor (1 ng/ml) and tumor necrosis factor-α (10 ng/ml) for 4 weeks with no medium changes. Morphological changes were evaluated under light microscopy. The percentages of fibroblast-like cells were calculated from two view fields at ×20 magnifications. The degree of the generation of fibroblast-like cells were scored as follows: 0, fibroblast-like cells <5%; 1, fibroblast-like cells 5% to 25%; 2, fibroblast-like cells 25% to 50%; 3, fibroblast-like cells >50%; 4, formation of a pile or a cluster in at least one view field. Statistical significance was evaluated by Spearman's rank correlation test.

There was a variation in the expression of NFκB1 mRNA among the RA patients. We next examined the relationship of the initial levels of NFκB1 mRNA in RA bone marrow CD34+ cells with their capacity to differentiate into fibroblast-like cells. As shown in Figure 6, there was a significant correlation between the NFκB1 mRNA expression and the generation of fibroblast-like cells from bone marrow CD34+ cells upon stimulation with SCF, GM-CSF and TNF-α for 4 weeks in 12 RA patients. The data indicate that the enhanced expression of NFκB1 mRNA is important for the enhanced generation of fibroblast-like cells.

### Effect of TNF-α on the expression of mRNAs for various components of NFκB in bone marrow CD34+ cells

**Figure 7:**
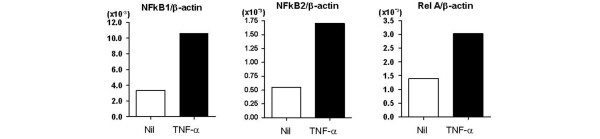
Effect of tumor necrosis factor (TNF)-α on the expression of mRNAs for nuclear factor (NF)κB1 (p50), NFκB2 (p52) and RelA (p65) in bone marrow CD34+ cells. Bone marrow CD34+ cells from healthy individuals were incubated in culture medium with or without TNF-α (10 ng/ml) for 24 hours. After the incubation, total RNA was isolated for evaluation of the expression of mRNAs for NFκB1, NFκB2, RelA and β-actin by real-time quantitative PCR. The data are expressed as the ratio of the mRNA copy numbers to those of β-actin. The data are representative of two different experiments.

Previous studies have demonstrated that TNF-α plays a critical role in the pathogenesis of RA [4]. It is possible, therefore, that the up-regulation of NFκB1 mRNA in bone marrow CD34+ cells might be secondary to the increased levels of TNF-α in the born marrow; experiments were carried out to test this possibility. Highly purified bone marrow CD34+ cells from healthy individuals were cultured in the presence of TNF-α (10 ng/ml) for 24 hours, after which the expression of mRNA for various components of NFκB was examined. As shown in Figure 7, treatment of bone marrow CD34+ cells with TNF-α upregulated not only the expression of NFκB1 (p50) mRNA, but that of NFκB2 (p52) mRNA and RelA (p65) mRNA. Since only the expression of NFκB1 mRNA, but not that of NFκB2 mRNA and RelA mRNA, was significantly upregulated in RA bone marrow CD34+ cells, the increased expression of NFκB1 mRNA in RA bone marrow CD34+ cells might not be accounted for simply by the increased levels of TNF-α in the bone marrow.

### Effect of silencing mRNA for NFκB1 on differentiation of RA bone marrow CD34+ cells into fibroblast-like cells upon stimulation with SCF, GM-SCF and TNF-α

**Figure 8:**
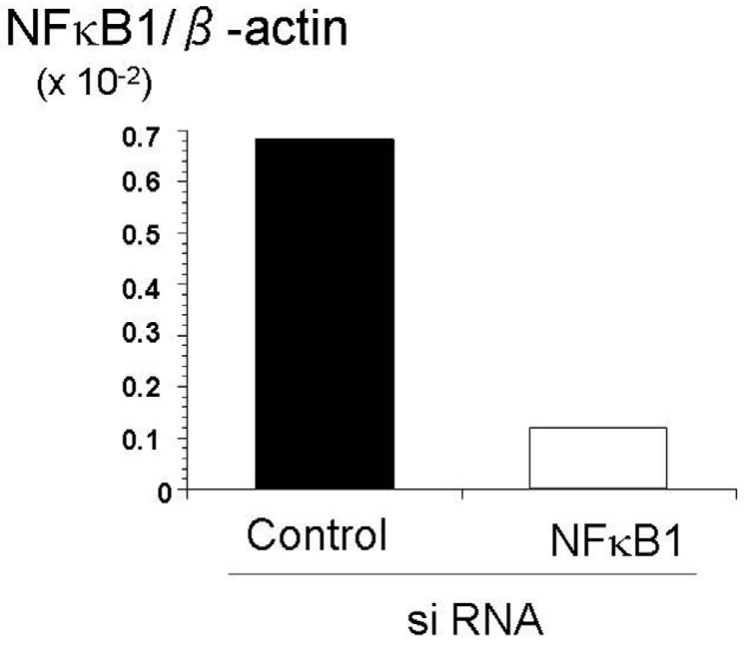
Silencing of nuclear factor (NF)κB1 mRNA in bone marrow CD34+ cells by small interfering RNA (siRNA) for NFκB1. Purified bone marrow CD34+ cells were transfected with siRNA for NFκB1 or a scrambled sequence control siRNA after a 24 hours incubation in culture medium with stem cell factor (10 ng/ml) and granulocyte-macrophage colony stimulating factor (1 ng/ml). After the transfection, the cells were further incubated for 48 hours in culture medium with stem cell factor and granulocyte-macrophage colony stimulating factor, and total RNA was isolated for evaluation of the expression of NFκB1 mRNA and β-actin mRNA by real-time quantitative PCR. The data are expressed as the ratio of the mRNA copy numbers to those of β-actin.

**Figure 9:**
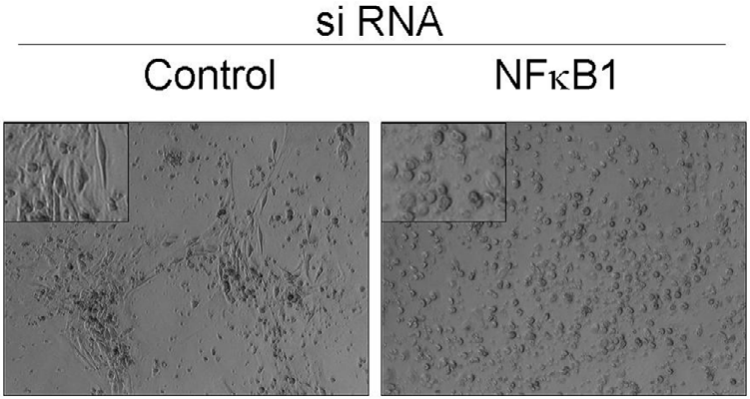
Inhibition of the generation of fibroblast-like cells by silencing nuclear factor (NF)κB1 mRNA in bone marrow CD34+ cells from patients with rheumatoid arthritis. Purified bone marrow CD34+ cells were transfected with small interfering RNA (siRNA) for NFκB1 or a scrambled sequence control, after which the cells were incubated in culture medium with stem cell factor (10 ng/ml), granulocyte-macrophage colony stimulating factor (1 ng/ml) and tumor necrosis factor-α (10 ng/ml) for 4 weeks with no medium changes. Morphological changes were observed under light microscopy (original magnification, ×20; inset, ×50 magnification). The data are representative of 12 different experiments.

**Figure 10:**
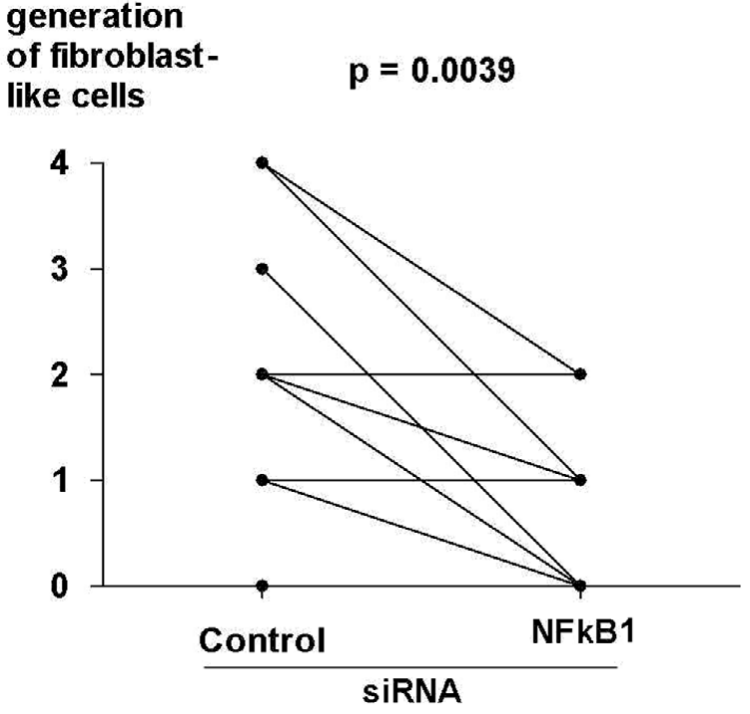
Inhibition of the generation of fibroblast-like cells by silencing nuclear factor (NF)κB1 mRNA in bone marrow CD34+ cells from patients with rheumatoid arthritis. Purified bone marrow CD34+ cells were transfected with small interfering RNA (siRNA) for NFκB1 or a scrambled sequence control siRNA, after which the cells were incubated in culture medium with stem cell factor (10 ng/ml), granulocyte-macrophage colony stimulating factor (1 ng/ml) and tumor necrosis factor-α (10 ng/ml) for 4 weeks with no medium changes. Morphological changes were observed under light microscopy. The percentages of fibroblast-like cells were calculated from two view fields at ×20 magnifications. The degree of the generation of fibroblast-like cells were scored as follows: 0, fibroblast-like cells <5%; 1, fibroblast-like cells 5% to 25%; 2, fibroblast-like cells 25% to 50%; 3, fibroblast-like cells >50%; 4, formation of a pile or a cluster in at least one view field. Statistical significance was evaluated by Wilcoxon's signed rank test.

**Figure 11:**
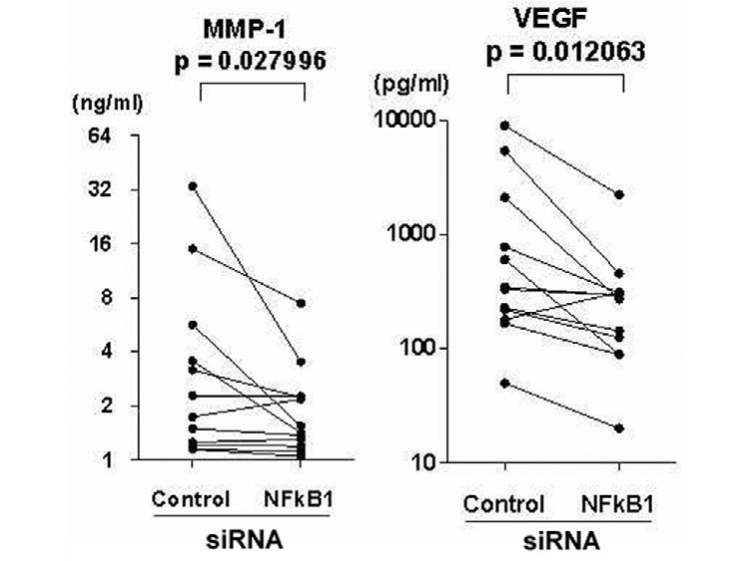
Suppression of the production of matrix metalloproteinase (MMP)-1 and vascular endothelial growth factor (VEGF) by silencing nuclear factor (NF)κB1 mRNA in bone marrow CD34+ cells from patients with rheumatoid arthritis. Purified bone marrow CD34+ cells from 12 patients with rheumatoid arthritis were transfected with small interfering RNA (siRNA) for NFκB1 or a scrambled sequence control siRNA, after which the cells were further incubated in culture medium with stem cell factor (10 ng/ml), granulocyte-macrophage colony stimulating factor (1 ng/ml) and tumor necrosis factor-α (10 ng/ml) for 4 weeks with no medium changes. After the incubation, the supernatants were harvested and assayed for MMP-1 and VEGF by ELISA. Statistical significance was evaluated by Wilcoxon's signed rank test.

**Figure 12:**
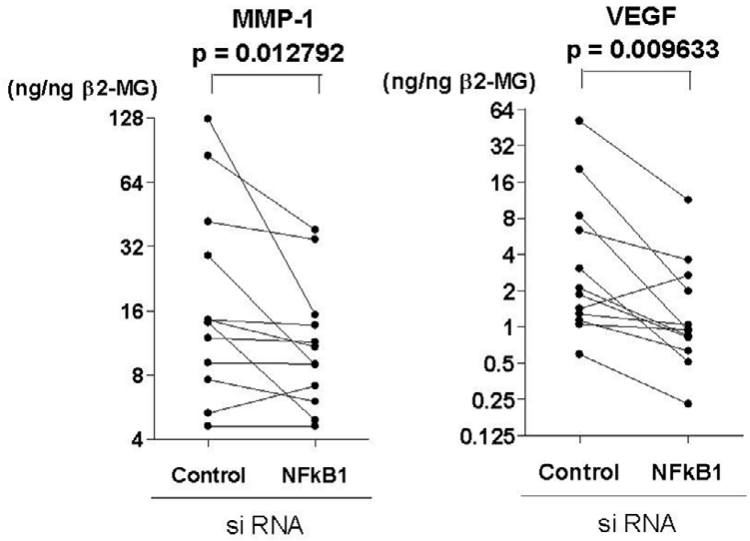
Suppression of the production of matrix metalloproteinase (MMP)-1 and vascular endothelial growth factor (VEGF) by silencing nuclear factor (NF)κB1 mRNA in bone marrow CD34+ cells from patients with rheumatoid arthritis. Purified bone marrow CD34+ cells from 12 patients with rheumatoid arthritis were transfected with small interfering RNA (siRNA) for NFκB1 or a scrambled sequence control siRNA, after which the cells were further incubated in culture medium with stem cell factor (10 ng/ml), granulocyte-macrophage colony stimulating factor (1 ng/ml) and tumor necrosis factor-α (10 ng/ml) for 4 weeks with no medium changes. After the incubation, the supernatants were harvested and assayed for MMP-1, VEGF and β_2_-microglobulin (β_2_MG) by ELISA. Statistical significance was evaluated by Wilcoxon's signed rank test.

**Figure 13:**
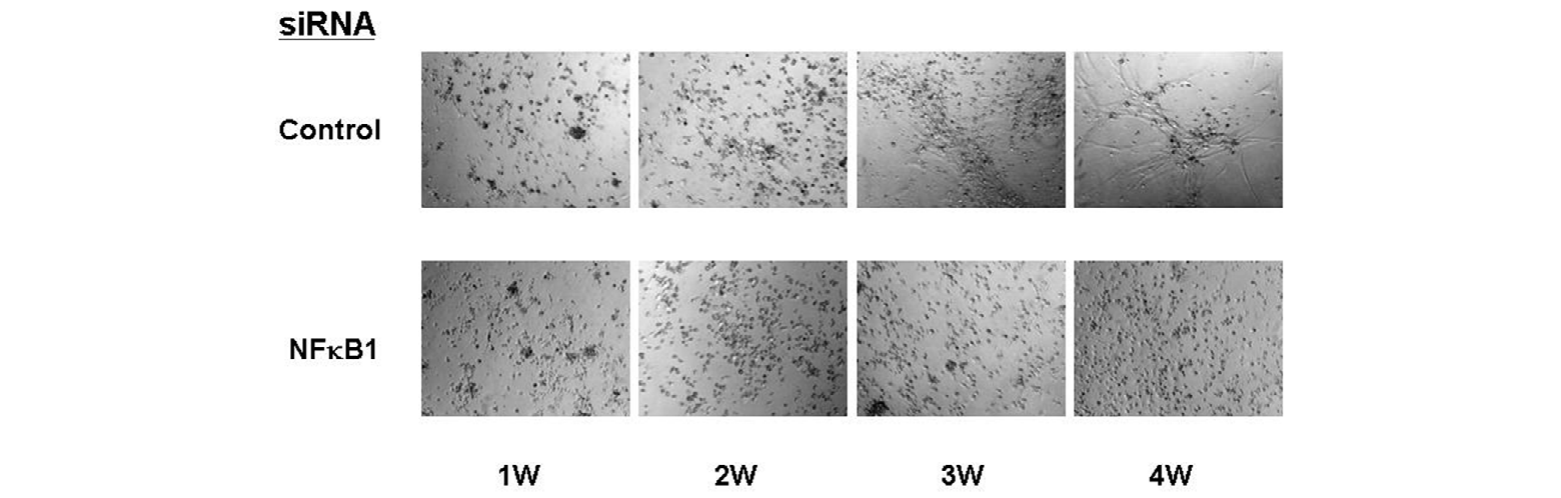
Time-kinetic effect of silencing nuclear factor (NF)κB1 mRNA in bone marrow CD34+ cells from patients with rheumatoid arthritis on the generation of fibroblast-like cells. Purified bone marrow CD34+ cells from patients with rheumatoid arthritis were transfected with small interfering RNA (siRNA) for NFκB1 or scrambled sequence control siRNA, after which the cells were further incubated in culture medium with stem cell factor (10 ng/ml), granulocyte-macrophage colony stimulating factor (1 ng/ml) and tumor necrosis factor-α (10 ng/ml) up to 4 weeks with no medium changes. After various periods of incubation (W, weeks), the morphological changes of the cells were observed under light microscopy. The data are representative of three different experiments.

**Figure 14:**
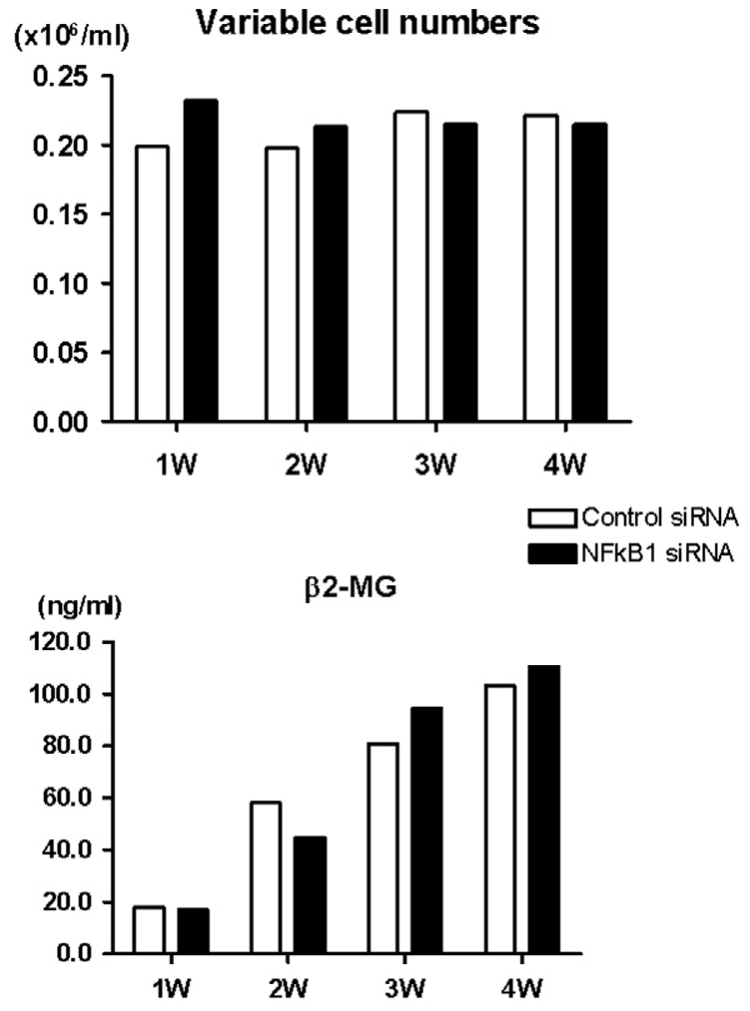
Time-kinetic effect of silencing nuclear factor (NF)κB1 mRNA in bone marrow CD34+ cells from patients with rheumatoid arthritis on the viable cell counts and the production of β_2_-microblobulin (β_2_MG). Purified bone marrow CD34+ cells from patients with rheumatoid arthritis were transfected with small interfering RNA (siRNA) for NFκB1 or scrambled sequence control siRNA, after which the cells were further incubated in culture medium with stem cell factor (10 ng/ml), granulocyte-macrophage colony stimulating factor (1 ng/ml) and tumor necrosis factor-α (10 ng/ml) up to 4 weeks with no medium changes. After various periods of incubation (W, weeks), the cells were counted and the quantities of β_2_MG in the culture supernatants were determined by ELISA. This is the same experment as shown in Figure 13. Data are representative of three different experiments.

We next examined whether silencing of NFκB1 (p50) mRNA in RA bone marrow CD34+ cells might correct their abnormal responses to TNF-α. As shown in Figure 8, treatment of bone marrow CD34+ cells with siRNA for NFκB1 reduced the expression of NFκB1 mRNA by approximately 80%. More importantly, reduction of NFκB1 mRNA markedly suppressed the generation of fibroblast-like cells from RA bone marrow CD34+ cells upon stimulation with SCF, GM-CSF and TNF-α (Figures 9 and 10). Accordingly, silencing of NFκB1 by siRNA significantly decreased the levels of MMP-1 and VEGF in culture supernatants of RA bone marrow CD34+ cells (Figure 11). Since bone marrow CD34+ cells proliferate in response to SCF, GM-CSF and TNF-α, it was possible that differences in MMP-1 and VEGF might be a result of alteration in cell proliferation by NFκB1 siRNA. Previous studies disclosed that β_2_MG is produced by a number of cell types, including lymphocytes, myeloid cells, and tumor cells [7-9]. The production of β_2_MG generally correlates with cell proliferation [6-9]. In fact, the levels of β_2_MG in the culture supernatants paralleled the viable cell counts of bone marrow CD34+ cells stimulated with SCF, GM-CSF and TNF-α. Of note, silencing of NFκB1 also significantly decreased the ratios of MMP-1 and VEGF to β_2_MG (MMP-1/β_2_MG and VEGF/β_2_MG) in culture supernatants of RA bone marrow CD34+ cells (Figure 12). Consistently, whereas siRNA for NFκB1 inhibited the differentiation of RA bone marrow CD34+ cells stimulated with SCF, GM-CSF and TNF-α into fibroblast-like cells (Figure 13), it significantly influenced neither the viable cell numbers nor the levels of β_2_MG in the culture supernatants (Figure 14). These results confirm that the enhanced expression of NFκB1 mRNA in RA bone marrow CD34+ cells led to their abnormal capacity to differentiate into fibroblast-like cells producing MMP-1 upon stimulation with SCF, GM-CSF and TNF-α without affecting cell viability or proliferation. The data suggest, therefore, that the enhanced expression of NFκB1 mRNA in bone marrow hematopoietic stem cells might play a pivotal role in the pathogenesis of RA.

## Discussion

The importance of TNF-α in the pathogenesis of RA has been well appreciated. Thus, anti-TNF-α antibodies and soluble TNF receptors have been demonstrated to have beneficial effects in the treatment of RA [4]. On the other hand, increasing attention has been paid to the role of bone marrow abnormalities in the pathogenesis of RA. In this regard, we demonstrated that RA bone marrow CD34+ cells have abnormal capacities to respond to TNF-α and to differentiate into fibroblast-like cells producing MMP-1 [2]. It should be noted that NFκB plays an important role in signal transduction and expression of a variety of genes, including MMP-1, under the influence of TNF-α [3]. The results in the current study have demonstrated that the expression of mRNA for NFκB1 is increased in RA bone marrow CD34+ cells. Of note, the expression of NFκB1 mRNA was significantly correlated with that of NFκB1 protein. Moreover, the initial levels of NFκB1 mRNA in RA bone marrow CD34+ cells were correlated with their capacity to differentiate into fibroblast-like cells upon stimulation with TNF-α. The data suggest that the increased expression of NFκB1 mRNA might lead to constitutive overproduction of NFκB p50 molecules and thus result in abnormal responses to TNF-α of RA bone marrow CD34+ cells. Of note, bee venom and its major component melittin have been shown to display anti-arthritic effects through inactivation of NFκB [10]. Since bee venom and melittin delay and reduce nuclear translocation of the p50 subunit of NFκB but not p65 (RelA) [10], the importance of NFκB p50 rather than p65 in the pathogenesis of inflammatory arthritides has been underscored.

In the present study, significant numbers of RA patients were treated with MTX and oral steroids. However, there were no significant differences in the expression of NFκB1 mRNA in bone marrow CD34+ cells between RA patients receiving MTX or oral steroids and those who were not, although the expression of NFκB1 mRNA appeared to be lower in RA patients receiving these drugs. It is suggested, therefore, that administration of MTX and oral steroids might have made the differences in the expression of NFκB1 mRNA in bone marrow CD34+ cells between RA and OA less marked. On the other hand, the expression of NFκB1 mRNA in bone marrow CD34+ cells was not correlated with serum CRP levels in RA patients. The upregulation of NFκB1 mRNA in bone marrow CD34+ cells might not, therefore, be secondary to systemic inflammation, but may be a primary abnormality intrinsic to RA.

In the present study, the expression of mRNA for RelA (p65) appeared to be decreased in RA bone marrow CD34+ cells compared with that in OA bone marrow CD34+ cells, although this decrease did not reach statistical significance. Of note, a previous study demonstrated that embryonic fibroblasts from RelA-deficient mice are defective in the TNF-α mediated induction of mRNAs for IκBα [11]. Moreover, in RelA deficient fibroblasts, IκBβ protein was absent, presumably due to the decreased stability of IκBβ mRNA [11]. Since IκB plays an important role in inhibition of translocation of NFκB into the nucleus, the decrease in RelA mRNA might result in enhanced activation of NFκB related genes through upregulation of the translocation of NFκB. It is suggested, therefore, that the decreased expression of RelA mRNA in RA bone marrow CD34+ cells might also contribute to abnormal response to TNF-α.

It is possible that the upregulation of NFκB1 mRNA in bone marrow CD34+ cells might be secondary to the increased levels of TNF-α in the bone marrow. In fact, the treatment of bone marrow CD34+ cells from healthy individuals with TNF-α resulted in the increased expression of NFκB1 mRNA. However, TNF-α also enhanced the expression of mRNAs for NFκB2 and RelA in bone marrow CD34+ cells from healthy individuals. Of note, the expression of RelA mRNA appeared to be rather decreased in RA bone marrow CD34+ cells as mentioned above. Taken together, these data strongly suggest that the enhanced expression of NFκB1 mRNA might not be due simply to the increased levels of TNF-α in the bone marrow. Further studies to explore the mechanism of abnormal expression of NFκB1 mRNA in bone marrow CD34+ cells would be important for delineation of the pathogenesis of RA.

The role of the enhanced expression of NFκB1 mRNA in RA bone marrow CD34+ cells in their abnormal responses to TNF-α was further confirmed by the experiments of selective silencing of NFκB1 mRNA. Reduction of NFκB1 mRNA in RA bone marrow CD34+ cells by transfection of siRNA for NFκB1 markedly suppressed the generation of fibroblast-like cells as well as the production of MMP-1 and VEGF under the influence of TNF-α without affecting the viability or the capacity to produce β_2_MG. These results indicate that upregulation of NFκB1 mRNA expression leads to the enhanced responses of RA bone marrow CD34+ cells to TNF-α. Thus, the enhanced NFκB1 mRNA expression might be a critical defect in RA bone marrow CD34+ cells.

Autologous hematopoietic stem cell transplantation (HSCT) has been used to treat severe RA in limited case reports [12,13]. However, a study with large numbers of patients has disclosed that recurrence of RA is frequent in patients who received autologous HSCT [14,15]. Frequent recurrence after autologous HSCT for RA suggests that abnormalities in bone marrow stem cells might persist after the treatment [16,17]. It is possible that the enhanced expression of NFκB1 mRNA might be closely related with such abnormalities in bone marrow stem cells, although further studies are required to confirm this point. It would also be important to explore whether there might be another transcription factor that could be inhibited without suppressing the differentiation of bone marrow CD34+ cells into fibroblast-like cells in order to confirm the importance of NFκB1 mRNA expression in the pathogenesis of RA.

## Conclusion

The present study has revealed the enhanced expression of NFκB1 mRNA in RA bone marrow CD34+ cells as possible intrinsic abnormalities in bone marrow, resulting in abnormal responses to TNF-α. Further studies to delineate the mechanisms for the abnormal NFκB1 mRNA expression would be important for a complete understanding of the pathogenesis and etiology of RA.

## Abbreviations

β_2_MG = β_2_-microglobulin; ELISA = enzyme-kinked immunosorbent assay; GM-CSF = granulocyte-macrophage colony stimulating factor; HSCT = hematopoietic stem cell transplantation; MFI = mean fluorescence intensity; MMP = matrix metalloproteinase; MTX = methotrexate; NFκB = nuclear factor kappa B; OA = osteoarthritis; PCR = polymerase chain reaction; PE = phychoerythrin; RA = rheumatoid arthritis; SCF = stem cell factor; siRNA = small interfering RNA; TNF-α = tumor necrosis factor-alpha; VEGF = vascular endothelial growth factor.

## Competing interests

The authors declare that they have no competing interests.

## Authors' contributions

SH designed the study, and participated in experimental procedures, collection, analysis, and interpretation of data, and manuscript preparation. YM and NC contributed to analysis and interpretation of data. TT, HY, and TO contributed to collection and analysis of data. All authors read and approved the final text before submission of the manuscript.

## Figures and Tables

**Table 1:** Primer sequences used in real-time quantitative PCR for analysis of mRNA for various nuclear factor κB components

Gene product (GenBank accession no.)	Primer sequences	Nucleotides
NFκB1 [M58603]	Forward: 5'-TCC ACA AGG CAG CAA ATA GA-3'	3,125–3,144
	Reverse: 5'-GGG GCA TTT TGT TGA GAG TT-3'	3,244–3,263
		
NFκB2 [NM_002502]	Forward: 5'-TTC TGA AGG CTG GTG CTG AC-3'	2,220–2,239
	Reverse: 5'-AGT GAG GTC AAG AGG CGT GT-3'	2,352–2,371
		
RelA [NM_021925]	Forward: 5'-GAA GAA GAG TCC TTT CAG CG-3'	1,011–1,030
	Reverse: 5'-GGG AGG ACG TAA AGG GAT AG-3'	1,116–1,135
		
β-actin [X00351]	Forward: 5'-GCA AAG ACC TGT ACG CCA AC-3'	910–929
	Reverse: 5'-CTA GAA GCA TTT GCG GTG GA-3'	1,150–1,169
